# Comparison of broad range 16S rDNA PCR and conventional blood culture for diagnosis of sepsis in the newborn: a case control study

**DOI:** 10.1186/1471-2431-9-5

**Published:** 2009-01-19

**Authors:** Tonje Reier-Nilsen, Teresa Farstad, Britt Nakstad, Vigdis Lauvrak, Martin Steinbakk

**Affiliations:** 1 Department of Pediatrics Akershus University Hospital, N-1478 Lørenskog, Norway; 2 University of Oslo, Akershus Faculty Division, N-1478 Lørenskog, Norway; 3 Department of Microbiology Akershus University Hospital, N-1478 Lørenskog, Norway

## Abstract

**Background:**

Early onset bacterial sepsis is a feared complication of the newborn. A large proportion of infants admitted to the Neonatal Intensive Care Unit (NICU) for suspected sepsis receive treatment with potent systemic antibiotics while a diagnostic workup is in progress. The gold standard for detecting bacterial sepsis is blood culture. However, as pathogens in blood cultures are only detected in approximately 25% of patients, the sensitivity of blood culture is suspected to be low. Therefore, the diagnosis of sepsis is often based on the development of clinical signs, in combination with laboratory tests such as a rise in C – reactive protein (CRP). Molecular assays for the detection of bacterial DNA in the blood represent possible new diagnostic tools for early identification of a bacterial cause.

**Methods:**

A broad range 16S rDNA polymerase chain reaction (PCR) without preincubation was compared to conventional diagnostic work up for clinical sepsis, including BACTEC blood culture, for early determination of bacterial sepsis in the newborn. In addition, the relationship between known risk factors, clinical signs, and laboratory parameters considered in clinical sepsis in the newborn were explored.

**Results:**

Forty-eight infants with suspected sepsis were included in this study. Thirty-one patients were diagnosed with sepsis, only 6 of these had a positive blood culture. 16S rDNA PCR analysis of blinded blood samples from the 48 infants revealed 10 samples positive for the presence of bacterial DNA. PCR failed to be positive in 2 samples from blood culture positive infants, and was positive in 1 sample where a diagnosis of a non-septic condition was established. Compared to blood culture the diagnosis of bacterial proven sepsis by PCR revealed a 66.7% sensitivity, 87.5% specificity, 95.4% positive and 75% negative predictive value. PCR combined with blood culture revealed bacteria in 35.1% of the patients diagnosed with sepsis. Irritability and feeding difficulties were the clinical signs most often observed in sepsis. CRP increased in the presence of bacterial infection.

**Conclusion:**

There is a need for PCR as a method to quickly point out the infants with sepsis. However, uncertainty about a bacterial cause of sepsis was not reduced by the PCR result, reflecting that methodological improvements are required in order for DNA detection to replace or supplement traditional blood culture in diagnosis of bacterial sepsis.

## Background

A large proportion of infants admitted to neonatal intensive care units (NICUs) present with signs of sepsis. In 2004, 130 patients or 35.4% of all newborns admitted to the NICU at Akershus University Hospital (Ahus), Norway, were treated with broad-spectrum systemic antibiotics for suspected bacterial sepsis. However, in only a small subset of the treated patients was a pathogen actually identified. Several studies have previously tried to find a clear-cut definition of sepsis in neonates, based on a combination of clinical signs and laboratory parameters [1-3]. However, diagnosing neonatal sepsis is difficult since being exposed to known risk factors for sepsis [4-7] is not a necessity, clinical signs are often vague, and laboratory parameters are unspecific. Conventional blood culture is considered the gold standard in the etiological diagnosis of neonatal bacterial sepsis [1,8]. However, obtaining sufficiently large amounts of blood for culture from neonates are often difficult [9-12], and it often takes 48–72 hours to obtain a preliminary positive result. Elevation of C – reactive protein (CRP) has been a useful marker of sepsis in many studies [1,2]. Initiation of broad-spectrum systemic antibiotic treatment is based only on the suspicion of sepsis since no early definitive diagnostic test is yet available. The clinician accepts some over-treatment because of the high risk of mortality if sepsis is left untreated. Two well-documented studies have shown that relevant pathogens were detected in about 25% of pediatric patients admitted to intensive care units [13,14]. Therefore, if no pathogenic bacterial agent is detected, the diagnosis of sepsis is based on the development of clinical signs only, often in combination with a rise in CRP [11,15].

Detection of bacterial DNA in blood samples of neonates is suggested to represent a rapid and sensitive supplement to blood culture in diagnosing bacterial sepsis in neonates [5,14,16]. However, at present there are no standardised clinically evaluated methods available for detection of bacterial DNA in blood samples from neonates. The main aim of this study was to compare a broad range 16S rDNA PCR done on whole blood samples without prior enrichment to conventional BACTEC Peds PLUS/F blood culture for detecting bacterial DNA in blood samples from infants with suspected sepsis.

In addition, the relationship between known risk factors, clinical signs, and laboratory parameters in suspected neonatal sepsis and the diagnosis of sepsis at discharge from the NICU were explored. Since the suspicion of sepsis is based on a number of known risk factors, clinical signs and laboratory markers, it would be helpful to identify a sign or marker that could predict the diagnosis of sepsis.

## Methods

### Patients

Infants with a birth weight (BW) > 1000 grams admitted to the NICU at Akershus University Hospital with suspected sepsis during their first week of life were included. All infants included in the study were treated with systemic antibiotics. The regional committee for medical ethics in science in Eastern Norway (REK1) approved the study and a written parental consent was obtained before inclusion.

Fifty-two infants were eligible for the study. Four of these patients were later not included in the analysis because of incomplete registration forms (3 infants) or missing samples for blood culture or PCR (1 infant).

### Study design

This project was carried out as a prospective non-randomised study. The clinical and laboratory variables registered at admittance are listed in Table 1 and Table 2.

**Table 1:** Comparison of clinical and laboratory parameters on admission in patients with sepsis (Sepsis) and patient with other non-infectious conditions (Not sepsis)

**Maternal parameters**	**Sepsis (n = 31)**	**Not sepsis (n = 17)**	**p**
Maternal fever > 38°C	10/31	5/17	0.25

Maternal antibiotics	10/31	0/17	0.00

Premature rupture of membranes	11/31	4/17	0.18

**Infant parameters**			

Gestational age (GA) < 37 weeks	3/31	6/17	0.03

Irritability	11/31	1/17	0.02

Fever > 38°C	7/31	5/17	0.23

Heart rate > 160 beats per minute	6/31	3/17	0.30

Respiratory rate > 60 per minute	11/31	5/17	0.23

Apgar score at 5 min < 8	3/31	7/17	0.01

Feeding difficulties	6/31	0/17	0.06

Capillary refill time > 3 seconds	7/31	3/17	0.27

Oxygen saturation (SaO_2_) < 88%	6/31	4/17	0.27

BE (base excess) < -5	0/31	5/17	0.00

Leukocyte count < 5	1/31	0/17	0.64

Platelets < 100	2/31	1/17	0.45

Blood glucose < 2,5 mmol/l	4/31	2/17	0.35

CRP mg/l	47.0 ± 33.8	8.5 ± 2.5	0.00

Highest CRP mg/l	66.8 ± 36.7	9.1 ± 2.7	0.00

**Table 2:** Comparison of clinical and laboratory parameters in patients with proven sepsis (sepsis with positive blood culture and/or detection of bacterial DNA in blood) and patients with non-proven sepsis (no bacterial agent found)

**Maternal parameters**	**Proven sepsis**	**Non-proven sepsis**	**p**
Maternal fever > 38°C	1/11	10/20	0.02

Maternal antibiotics	2/11	8/20	0.15

Premature rupture of membranes	3/11	8/20	0.25

Normal vaginal delivery	5/11	5/20	0.16

**Infant parameters**			

Gestational age (GA) < 37 weeks	0/11	2/20	0.40

Irritability	6/11	6/20	0.13

Fever > 38°C	2/11	5/20	0.32

Heart rate >160 beats per minute	2/11	3/20	0.37

Respiratory rate > 60 per minute	3/11	8/20	0.21

Apgar at 5 min < 8	1/11	3/20	0.40

Feeding difficulties	2/11	4/20	0.32

Capillary refill time > 3 seconds	1/11	7/20	0.11

Oxygen saturation (SaO2) < 88%	3/11	3/20	0.26

BE < -5	0/11	0/20	1.0

Leukocyte count < 5	0/11	1/20	0.47

Platelets < 100	2/11	0/20	0.12

Blood glucose < 2,5 mmol/l	0/11	3/20	0.25

CRP mg/l	49.3 ± 41.3	42.7 ± 29.1	0.60

Highest CRP mg/l	71.7 ± 41.6	70.3.6 ± 35.5	0.92

### Microbial analyses

A minimum of 1 ml full blood for conventional BACTEC Peds PLUS/F blood culture, and 1–2 ml EDTA-blood for 16S rDNA PCR were obtained by standard sterile procedures before starting general systemic antibiotic treatment. Only one blood culture bottle was routinely drawn from each patient. The bottles for culturing were immediately incubated. The EDTA-blood samples for PCR were blinded and stored in room temperature for until 72 hours, divided into plasma and cell fractions, and were then stored at -70°C before analysis.

### PCR reactions and detection limits

PCR reactions were set up to amplify bacterial DNA using the primer 5'TGAAGAGTTTGATCATGGCTCAG combined with either primer 5'AAGGAGGTGATCCAACCG, 5'TCGTTGCGGGACTTAACC or 5'TACCGCGGCTGCTGGCA. The primers react with highly conserved regions of the bacterial 16S rRNA gene to provide PCR products of approximately 1500 basepairs (PCR 1), 1100 basepairs (PCR 2) and 500 basepairs (PCR 3), respectively [17]. All primers were produced at Eurogentec, Belgium, and are routinely used in our laboratory for 16S rRNA based identification of unknown isolates. Each PCR reaction (50 μl) consisted of 1 × Amplitaq Gold buffer (Applied Biosystems) supplemented with 2.5 U Amplitaq Gold Low DNA enzyme (Applied Biosystems), 2 mM MgCl_2_, 0.2 mM dNTP (Roche) 20 μl template and PCR grade water (Roche). Cycling conditions included a 5 minute denaturing step at 94°C followed by 30 to 40 cycles of 20 seconds at 94°C, 20 seconds at 58°C and 60 seconds at 72°C. Detection limit in cfu/ml (colony forming units, cfu) for each PCR was established in triplicate reactions of extracts from blood samples spiked with *Staphylococcus aureus (S.aureus) *and *Escherichia coli (E.Coli) *at known concentrations (cfu/ml). The spiked blood samples were also divided in plasma and cell fraction and stored at -70°C. Nucleic acid was extracted from duplicates of 200 μl samples of both fractions using the Qiagen Blood kit. DNA from each extraction was recovered with 100 μl PCR grade water (Roche). The limit of detection was set to the number of cfu/ml of blood where all triplicates were positive. For PCR1 40 cycles of PCR could be used to detect 10^3 ^cfu/ml of both *E.coli *and *S.aureus*. For PCR2 we were unable to find conditions where 10^4 ^cfu/ml or less could be detected without also observing contaminants in the non-spiked controls. For PCR3 35 cycles of PCR could be used to detect 10^4 ^cfu/ml, however additional bands appeared when increasing the cycle numbers and dominated in samples with 10^3 ^cfu/ml or less.

The EDTA blood from newborns with signs of sepsis were analysed by blinded PCR using conditions PCR 1 and PCR 3 established for the spiked blood samples. PCR results were considered positive when visible PCR products of the correct size were found in at least duplicate reactions in runs with DNA isolated from either the plasma or the cell fraction. PCR results were considered negative when no visible PCR products of correct size were found. Results were considered inconclusive when single positive runs could not be repeated [18].

Attempts to sequence the positive PCR products were made using the PCR primers, standard Big Dye terminator cycle sequencing by capillary gel electrophoresis (Applied Biosystems). If a sequence was established, an identity match to the region between the primers was then searched in the Bio-Informatic Bacterial identification database (BiBi, Lyon France).

### Data analysis

Included patients were allocated into one of two groups, S (Sepsis) and nS (not Sepsis), based on the diagnosis at discharge. The following patients in the S group were discharged from the NICU with the diagnosis of sepsis: infants with suspicion of sepsis and an elevated CRP, development of clinical signs consistent of sepsis, a marked CRP-rise, or detection of a relevant pathogen in blood culture. Patients in the S group were further divided into two groups, "proven sepsis" (with detection of a pathogen bacteria in blood culture or by PCR), and "non-proven sepsis" (without detection of a pathogen with either method). Risk factors, clinical signs and laboratory parameters were compared between the two groups S and nS, and between "proven sepsis" and "non-proven sepsis". Nonparametric test for independent samples (the Mann-Whitney Test) was used to compare linear variables between groups. For categorical variables the Fisher exact test was used. A p-level of < 0.05 was considered statistically significant. The statistical program SPSS version 12.01 was used for data analysis.

## Results

Forty-eight infants with suspected sepsis were included in this study. At discharge from the NICU, 31 patients were diagnosed with sepsis, including both bacterial proven and non-proven sepsis, and 17 infants were discharged with a diagnosis of other non-infectious diseases. Of newborns diagnosed with sepsis, six infants had a positive blood culture and nine had a positive PCR.

### Comparison of 16S rDNA PCR and blood culture

The results are presented in Table 3 and Table 4.

**Table 3:** Comparison of broad range 16 S rDNA and conventional Bactec blood culture

	**Number of patients**	**Broad Range 16 S rDNA PCR**	
**Sepsis**		Negative	Positive

Bactec +	6#	2*	**4**

Bactec -	25	**20**	5

**Not sepsis**			

Bactec +	0	0	**0**

Bactec -	17	**16**	1

# Culture from one patient grew four organisms (Klebsiella spp, Escherichia coli, a viridans Streptococcus and Staphylococcus aureus), culture from another patient grew two organisms (an unnamed viridans Streptococcus and Streptococcus gordomitis). Both had a positive PCR-result and were diagnosed with sepsis based on additional clinical and laboratory findings.

* One of these patients had an inconclusive PCR.

**Table 4:** Microbiological results of Broad Range 16 S rDNA and conventional Bactec blood culture

**Broad Range 16 S rDNA PCR**	**Conventional Bactec blood culture**
Positive	beta-haemolytical Streptococcus gr B

Positive	Negative

Positive	beta-haemolytical Streptococcus gr B

Inconclusive	Staphylococcus lugduensis

Positive	Streptococcus gordomitis and an unnamed viridans streptococcus (Mix)

Positive/Haemophilus parainfluenzae	Negative

Positive	Negative

Staphylococcus aureus	Negative

Positive	viridans streptococcus

Negative	Klebsiella, Escherichia coli, Staphylococcus aureus and viridans streptococcus (Mix)

Positive	Negative

Six of the infants in this study had a positive blood culture result. Two of them had mixed bacteria in their blood culture. A pathogenic bacterium was detected in blood cultures from 6 of 48 patients, counting for 19.4% (6/31) of the patients diagnosed with sepsis in this study. Ten patients had a positive PCR however, one was diagnosed with asphyxia and not sepsis, therefore 29.0% (9/31) of patients were diagnosed with sepsis. Correlation between the results from PCR and blood culture was not obvious. One patient with a positive blood culture had a negative PCR result, and another patient had an inconclusive PCR result. Six patients had a positive PCR in spite of a negative blood culture. Compared to blood culture the diagnosis of bacterial sepsis in the newborn by PCR revealed 66.7% sensitivity, 87.5% specificity, 95.4% positive and 75% negative predictive value. Altogether, PCR and/or blood culture detected bacteria in 35.5% (11/31) of the patients with the diagnosis of sepsis. In our study, compared to the diagnosis of sepsis, PCR had 29.0% sensitivity, 94.1% specificity, 90% positive and 44.4% negative predictive values.

### Risk factors, clinical signs and laboratory parameters in S (sepsis) versus nS (not sepsis)

Results are presented in Table 1.

Known risk factors [1,19] such as maternal fever with temp. > 38°C at delivery and ruptured membranes > 12 hours before delivery were not significantly higher in the group with sepsis. Neither did discolouration of amniotic fluid, instrumental intervention during birth or Apgar score of < 7 at 1 min. differ significantly between the two groups (data not shown in Table 1). Apgar scores < 8 at 5 min and infants with gestational ages < 37 weeks were significant higher in the non-septic group. Clinical signs of sepsis, such as fever in the newborn (defined as rectal temp. > 38°C), and tachycardia (defined as heart rate > 160 beats per minute), did not correlate significantly with either of the two groups. Furthermore, respiratory signs seemed to be particularly unspecific, since tachypnoea with respiratory rate (RR) > 60/min., abnormal respiratory pattern, oxygen saturation (SaO_2_) < 88% and abnormal thoracic x-ray (data not shown), did not correlate with either of the two groups. Irritability and feeding difficulties seemed to be the only clinical signs to be trusted in our study when diagnosing sepsis in the newborn. Infants in the not sepsis group presented more often with base excess < -5 on admittance to the NICU. Patients in the sepsis group had mean CRP concentrations of 47.0 (confidence interval (CI) 13.2–80.8) mg/L at admittance to the NICU, and 66.8 (CI 30.1–103.5) mg/L as highest CRP during treatment. The non-sepsis group had mean CRP concentrations of 8.5 (CI 6–11) mg/L at admittance to the NICU, and 9.1 (CI 6.4–11.8) mg/L as highest CRP during treatment.

### Risk factors, clinical signs and laboratory parameters in "bacterial proven sepsis" versus "non-proven sepsis"

Results are presented in Table 2.

Maternal fever at delivery was more commonly seen in infants in whom a pathogenic agent was not detected. Although instrumental intervention, discolouration of amniotic fluid and a low Apgar score tended to be overrepresented in infants with non-proven sepsis, no other risk factors analysed discriminated significantly between the two groups. Capillary filling time > 3 sec. together with respiratory rate > 60 breaths per min. were observed more often in non-proven sepsis, while irritability was seen more often in infants with proven sepsis, although none of these findings were significant. No laboratory parameters analysed differed significantly between the two groups, but an abnormal platelet level seemed to be more common in infants with proven sepsis.

## Discussion

### Comparison of 16S rDNA PCR and blood culture for detecting bacteria in newborns with signs of sepsis

In the present study the diagnosis of bacterial sepsis in the newborn by PCR revealed 66.7% sensitivity, 87.5% specificity, 95.4% positive and 75% negative predictive value. While neonatal sepsis was diagnosed in 31 out of the 48 patients enrolled in the study, a pathogenic bacterium was detected in the blood culture of only 19.4% of these patients. With the molecular method of broad range 16S rDNA PCR, the detection of bacteria improved to 29.0%. As the total number of patients was low this difference did not obtain statistical significance. Based on the criteria used for the diagnosis of sepsis, these two methods combined had a sensitivity of 35.5%.

Six patients tested positive for broad range bacterial PCR but had negative blood cultures. Five of these six patients were diagnosed with sepsis. The blood cultures may have been negative due to inadequate amount of blood drawn for optimal detection of bacteria [20-22]. Kellogg et al. [23-25] found that low-level bacteraemia (<10 cfu/ml) is far more common (up to 68%) in paediatric patients than previously believed. They concluded that it is necessary to collect up to 4.5% of the patient's blood volume (approximately 4 ml/kg) in at least two blood cultures to detect low concentrations of pathogens in the blood. However, as neonates are very sensitive to even small losses of blood, collecting more than 1–2 ml of blood is not an option for this group of patients. With the chosen PCR procedure, we observed a detection limit of 10^3^-10^4 ^cfu/ml in triplicate extracts of spiked blood samples, not allowing detection of low level bacteraemia. In one patient, blood culture was positive (*S.aureus*) with a concordant negative PCR result. Another patient had a coagulase-negative *Staphylococcus *in blood culture but an inconclusive PCR at both 35 and 40 cycles. Both of these patients were considered as having proven sepsis and the result could reflect the presence of a low level of live bacteria. Even if improving the detection limit, low level bacteraemia (<10 cfu/ml) in neonates will be difficult to identify by any method based on detection of bacterial DNA or growth. To our knowledge, there are no standardised, clinically evaluated tests available for the detection of pathogenic nucleic acid in blood samples of neonates. One available PCR based test for detection of pathogenic DNA in blood of patients with suspected sepsis is developed by Roche, the LightCycler SeptiFast system. The test detects a total of 25 pathogenic bacteria and fungi in blood samples, is standardised and commercially available in Europe. However, it is not evaluated for neonates, and a volume of blood of 3 ml is required.

Jordan et al [14,26,27] showed a higher level of agreement between the two methodologies when preincubation was performed before PCR testing. They used 200–500 microl. EDTA-fullblood preincubated at 37°C for 5 hours before PCR-testing, and found 96% sensitivity, 99.4% specificity, and 88.9% positive and 99.8% negative predictive values for PCR compared with the culturing of 0.5–1.0 ml full blood with BACTEC 9240. However, a drawback with this procedure is that only live bacteria, able to grow in blood culture bottles will be detected. In our study we omitted the enrichment step and this might explain the difference between the two methods compared to what was reported by Jordan et al [12,23,27]. We can not exclude that the six positive PCR-results without concordant culture-positive samples could also result from contamination. However, since five of these patients ended up with the diagnosis of sepsis based on clinical judgements and a rise in CRP, contamination seems less likely.

### Risk factors, clinical signs and laboratory parameters

The World Health Organization (WHO) has suggested simple clinical criteria for the diagnosis of sepsis [28]. These criteria are based on studies in children from Ethiopia, The Gambia, Papua New Guinea and The Philippines, less than 91 days of age, who were examined because of complaints indicating possible infection. These criteria can not be easily adapted to developed countries where infants seek earlier medical attention, while symptoms are still vague. In our study, irritability in the newborn was the clinical sign observed most often (p = 0.02) in infants with the diagnosis of sepsis. The absence of the other clinical criteria suggested by WHO could be explained by the small number of patients with serious bacterial illness in our study [29]. Maternal fever during labour (p = 0.02) was more common in infants with non-proven sepsis, which may be explained by the fact that mothers with fever at delivery were more often given prophylactic antibiotic therapy. CRP is the most commonly used marker for identifying neonates with sepsis [2,15]. It has previously been suggested that serial elevated CRP levels are more appropriate than a single CRP measurement in diagnosing sepsis [3]. Previously, authors have operated with different cut off points for CRP in the diagnosis of neonatal clinical septicemia [2,3]. In this study, we did not operate with any clear cut off points in CRP before starting treatment. In view of these statements, it seems quite unlikely that neonates in this study with serial normal CRP levels e.g. < 10 mg/L, did have sepsis.

## Conclusion

Different medical conditions may mimic signs of sepsis in newborns, especially stress during birth. In the present study, irritability, feeding difficulties and a marked rise in CRP were important in establishing the diagnosis of sepsis in the newborn. 16S rDNA PCR method used in this study increased the sensitivity in detecting bacterial DNA in newborns with signs of sepsis, although not significantly. PCR has potential as a method for earlier detection of bacteria but this technology needs to be further developed and improved. Blood culture is irreplaceable at present, since pure isolates are essential for antimicrobial drug susceptibility testing.

## Competing interests

The authors declare that they have no competing interests.

## Authors' contributions

TRN, TF, BN, and MS were responsible for the study design. VL modified, validated and performed the PCR analysis. TRN was responsible for the clinical study implementation. All authors participated in writing and/or reviewing of this manuscript.

## Pre-publication history

The pre-publication history for this paper can be accessed here:


